# Spontaneous Unusual Backflow from Duodenum to Biliary System in a Dog with Pancreatic Abscesses: A Case Study

**DOI:** 10.3390/ani15081089

**Published:** 2025-04-09

**Authors:** Robert Cristian Purdoiu, Sorin Marian Marza, Radu Lacatus, Lucia Bel, Lea Carisch, Patrick Kircher

**Affiliations:** 1 Laboratory of Radiology and Medical Imaging, Faculty of Veterinary Medicine, University of Agricultural Sciences and Veterinary Medicine, Calea Mănăștur 3-5, 400372 Cluj-Napoca, Romania; robert.purdoiu@usamvcluj.ro (R.C.P.); radu.lacatus@usamvcluj.ro (R.L.); luci.bel@usamvcluj.ro (L.B.); 2 Clinic for Diagnostic Imaging, Department of Diagnostics and Clinical Services, Vetsuisse Faculty, University of Zurich, Winterthurerstrasse 260, 8057 Zurich, Switzerlandpatrick.kircher@uzh.ch (P.K.)

**Keywords:** abscessing pancreatitis, barium sulfate, dog, duodenobiliary reflux, multimodal imaging, veterinary imaging

## Abstract

Spontaneous duodenobiliary reflux is a rare condition in veterinary medicine, previously documented in only one canine case. We report a second instance in a 3-year-old neutered male Yorkshire Terrier, detected incidentally during a barium contrast study of the gastrointestinal tract (GIT). Multimodal imaging, including radiography, computed tomography (CT), and ultrasonography, confirmed the reflux and identified abscessing pancreatitis as its cause—a previously unreported etiology in dogs. This case expands the understanding of duodenobiliary reflux in canines, identifies abscessing pancreatitis as a novel differential, and demonstrates the diagnostic value of advanced imaging. In conclusion, abscessing pancreatitis should be considered as a potential cause of duodenobiliary reflux in dogs, and the use of multiple imaging modalities is pivotal for accurate diagnosis and management of this unusual condition.

## Simple Summary

Duodenobiliary reflux, the retrograde flow of duodenal contents into the biliary system, is exceptionally rare in dogs, with only one prior reported case. This study describes a unique instance in a 3-year-old neutered male Yorkshire Terrier, identified incidentally during a barium sulfate gastrointestinal examination. Advanced imaging—radiography, computed tomography (CT), and ultrasonography—revealed pancreatic abscesses as the underlying cause, a novel etiology in veterinary medicine. Unlike human cases, where reflux often leads to infections or stone formation, this dog showed no immediate complications. This case underscores an unusual cause of duodenobiliary reflux and highlights the critical role of multimodal imaging in veterinary diagnostics.

## 1. Introduction

Duodenobiliary reflux, the spontaneous retrograde movement of duodenal contents into the biliary system, is well-documented in human medicine. It is often linked to sphincter of Oddi dysfunction, biliary-enteric fistulae, or post-surgical anatomical changes that compromise the one-way flow at the duodenal papilla [1,2]. Sphincter of Oddi dysfunction (SOD) in particular can permit reflux when the sphincter fails to prevent backflow, as demonstrated by increased duodenal-biliary reflux in patients with sphincter hypomotility [3]. Early human reports of duodenobiliary reflux date back to 1915, when barium sulfate reflux was observed during upper GIT examinations [4,5]. Barium sulfate is a non-ionic, insoluble contrast agent valued for its inertness and high radiopacity; unlike hyperosmolar iodinated contrast agents, barium does not induce significant sphincter spasm [6,7]. This property has made barium useful in revealing reflux phenomena in both humans and animals. In veterinary medicine, duodenobiliary reflux is exceedingly rare, with only two prior cases: one dog following duodenal resection for a perforated ulcer [8] and one cat after foreign body intestinal obstruction [9].

Here, we present a spontaneous case of duodenobiliary reflux in a dog, identified during a barium study and attributed to abscessing pancreatitis (an acute pancreatitis with abscess formation). This report introduces a novel veterinary etiology for duodenobiliary reflux, draws parallels to human pathophysiology, and emphasizes the diagnostic utility of multimodal imaging [10].

## 2. Materials and Methods

### 2.1. Case Details

A 3-year-old neutered male *Yorkshire Terrier* was presented to the Radiology Service at the University of Agricultural Sciences and Veterinary Medicine, Cluj-Napoca, Romania, with a five-day history of anorexia, vomiting, and lethargy. He weighed 5.0 kg and had an elevated body condition score (7/9, indicating overweight, was used Body Condition Score (BCS) from Association for Pet Obesity Prevention). Clinical examination found mild abdominal distension, abdominal muscle tension, and pain on palpation; pain was assessed using the short form composite measure pain score (CMPS-SF) as approximately 11/24. Initial laboratory tests showed neutrophilic leukocytosis and mild elevations in liver enzymes (alanine aminotransferase and alkaline phosphatase). A canine pancreas-specific lipase (cPL) test was strongly positive (>400 μg/L), supporting a diagnosis of pancreatitis. All procedures were performed with informed owner consent and adhered to institutional ethical guidelines. As this is a single-case report of a spontaneous presentation, no formal inclusion or exclusion criteria were applicable, or institutional Animal Ethics Committee approval was required.

### 2.2. Imaging Approach

Initial right lateral abdominal radiography (TUR-GRx, K&S Rontgenwerk, Bochum, Germany; 10, 40 kVp, 10 mAs) was performed to screen for gastrointestinal foreign bodies. A barium contrast study (10 mL/kg, 60% *w*/*v* emulsion) was conducted to assess gastrointestinal motility. Radiographs were taken at 30 min, 2 h, and then once daily for 3 consecutive days (40 kVp, 10 mAs) to monitor barium progression and clearance, with the first radiograph at 30 min post-contrast to assess initial gastric emptying and duodenal filling.

Forty-eight hours after barium administration, a contrast-enhanced helical CT scan of the abdomen was performed (Siemens Somatom Scope, Siemens Healthineers, Erlangen, Germany; 120 kVp, 110 mAs; 512 × 512 matrix; soft tissue kernel; 1 mm slice thickness; pitch 1.5), followed by multiplanar and 3D volumetric reconstructions.

At the same 48-h mark, transabdominal ultrasonography was carried out using an Esaote MyLab X7 unit with a 5–10 MHz convex transducer (B-mode, 8 MHz selected frequency, gain 50–60%). The ultrasonographic examination focused on the duodenum, pancreas, liver, and gallbladder to correlate with the radiographic and CT findings.

### 2.3. Treatment and Follow-Up

Supportive and medical therapy was initiated immediately after consultation. The dog received intravenous fluids (lactated Ringer’s, 60 mL/kg per day), antibiotics (amoxicillin-clavulanate, 20 mg/kg IV twice daily), and analgesia (meloxicam, 0.1 mg/kg SC once daily). Opioid analgesics were avoided prior to diagnosis to prevent further gastrointestinal motility depression, given the risk of ileus [11], after establishing the clinic diagnostic tramadol (5 mg/kg P.O) was associated with meloxicam for pain management. Despite aggressive medical management, the dog remained anorectic, and abdominal pain persisted. An exploratory laparotomy was performed on day 3 to assess pancreatic and abdominal cavities. In surgery, multiple small pancreatic abscesses were observed alongside pancreato-duodenal adhesions and a visibly inflamed, distended major duodenal papilla. No surgical resection or drainage of the abscesses was attempted due to their diffuse distribution and the concern for destabilizing the patient. The procedure primarily served a confirmatory and diagnostic purpose. Unfortunately, the dog’s condition deteriorated rapidly after surgery, with refractory vomiting, suspected sepsis, and worsening lethargy. Given the grave prognosis, the owners elected humane euthanasia on day 4. No post-mortem histopathology was performed per the owners’ wishes, which limited definitive pathological confirmation.

## 3. Results

**Figure 1:**
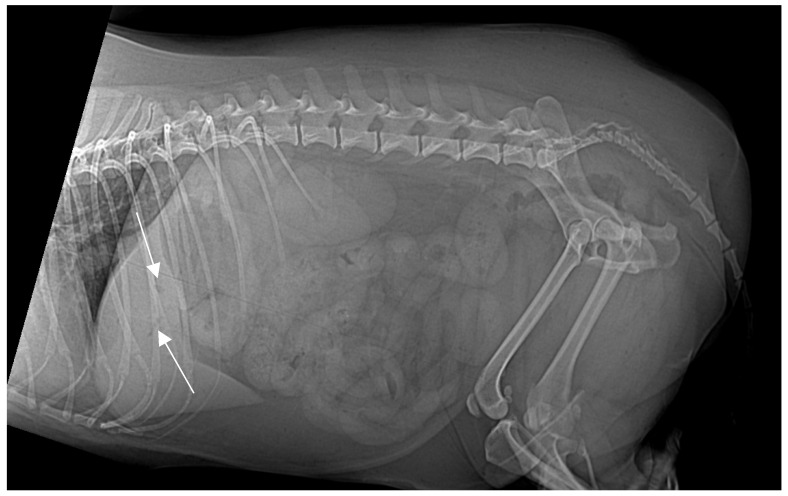
Right lateral abdominal X-ray projection. No foreign bodies identified. Small, tubular gas-opacity structures (white arrows) overlap the liver’s caudal margin cranial to the stomach and pyloric antrum, suggesting pneumocholecystitis (gas within the biliary system).

On presentation, the patient was mildly obese (BCS 7/9) and exhibited abdominal discomfort. Initial survey radiographs showed no evidence of gastrointestinal obstruction or radiopaque foreign material. However, the lateral abdominal view revealed several small, tubular gas opacities superimposed on the caudal aspect of the liver silhouette, cranial to the stomach, and pyloric antrum, suggestive of gas within the biliary tree (pneumocholecystitis) (Figure 1).

**Figure 2:**
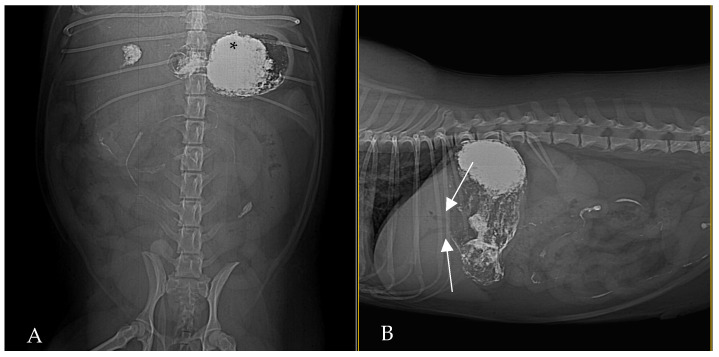
Ventrodorsal (**A**) and lateral (**B**) abdominal radiograph, 30 min post-barium administration. The stomach is filled with barium (asterisk), and small amount of barium is visible in small intestines. There is no evidence of biliary reflux at this time. Gas persists in biliary tree ((**B**), white arrows).

At 30 min post-barium, ventrodorsal and lateral radiographs demonstrated a barium-filled stomach with evident rugal folds and a small amount of barium that beginning to pass in the small intestines, with no biliary reflux of barium evident at that time (Figure 2). Gas was persistent in the biliary tree, appearing slightly more pronounced than on the initial radiograph.

**Figure 3:**
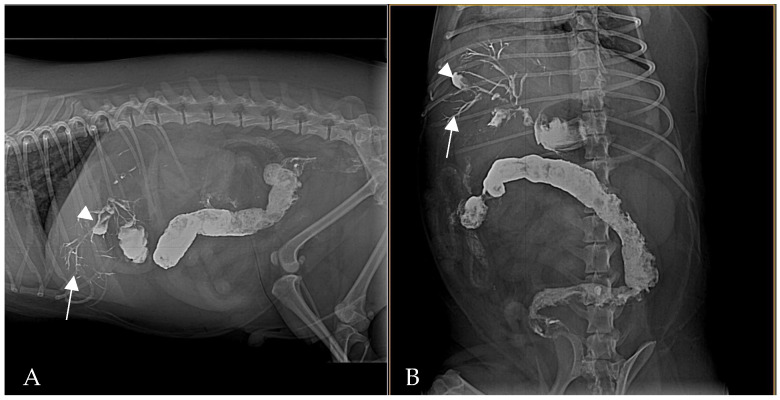
Right lateral (**A**) and ventrodorsal (**B**) abdominal radiography at 2 h post-barium. Barium is visible outlining the biliary tree (white arrows) and is beginning to fill the gallbladder (white arrowhead). Residual contrast is also present in the stomach and intestines, and fecal matter mixed with barium is seen in the colon.

**Figure 4:**
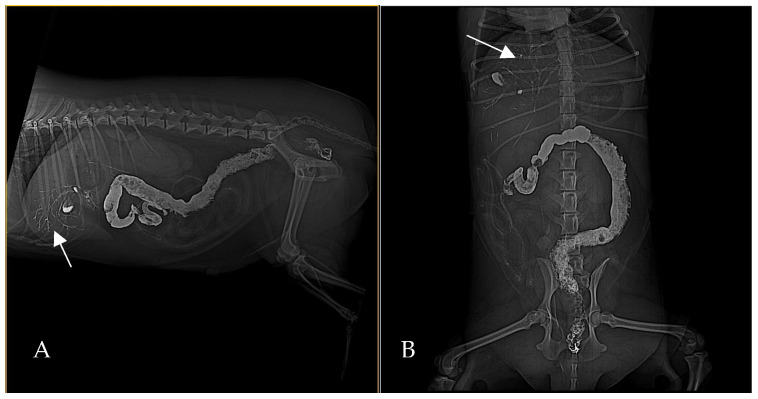
Right lateral (**A**) and ventrodorsal (**B**) abdominal radiographs, 48 h post-barium. Barium contrast is mildly persistent in the biliary tree (arrows), and the contents of the colon show increased opacity due to a large amount of residual barium. Most of the contrast has cleared by this time, except for that in the biliary system and colon.

By 2 h post-barium (Figure 3) administration, radiography demonstrated biliary tree and gallbladder had become opacified with barium, indicating retrograde flow of contrast from the duodenum into the biliary ducts. This reflux was even more conspicuous in subsequent images: at 24 h post-contrast, the branching biliary tree was fully outlined by hyperattenuating contrast medium, and the gallbladder lumen was coated with barium. Concurrently, by 24 h a substantial amount of barium had reached the transverse and descending colon, increasing the opacity of the colonic contents. Barium residues cleared gradually over the next 72 h, with most of the contrast eliminated by day 3 (Figure 4).

**Figure 5:**
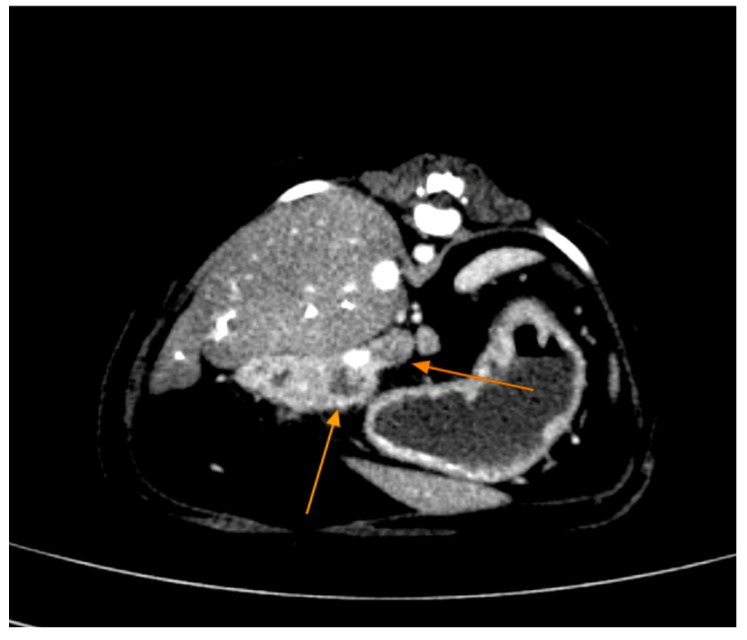
Abdominal contrast CT (axial plane) shows an enlarged, irregular pancreas with multiple hypoattenuating abscesses (red arrows) within the pancreatic tissue. Peripancreatic fat stranding is evident as hazy areas around the pancreas. The surrounding organs show mild reactive changes.

**Figure 6:**
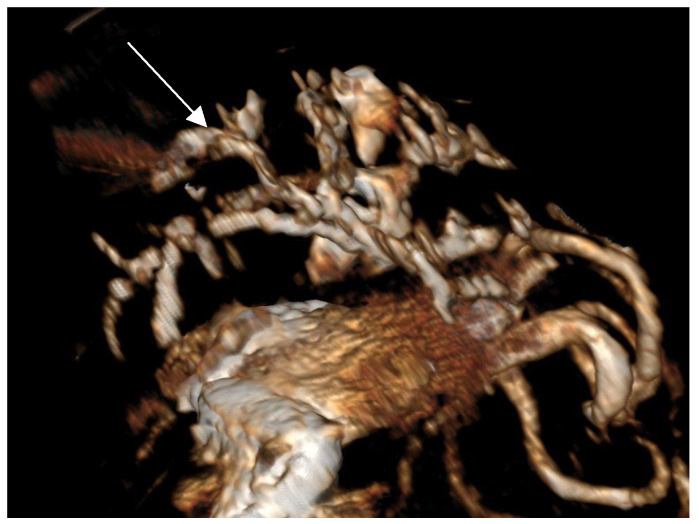
Volumetric 3D reconstruction of the abdomen (soft tissue subtracted) highlighting the distribution of barium sulfate (bright white) within the biliary tree (arrows). The intrahepatic and extrahepatic bile ducts and gallbladder (GB) are opacified by barium, confirming duodeno-biliary reflux.

**Figure 7:**
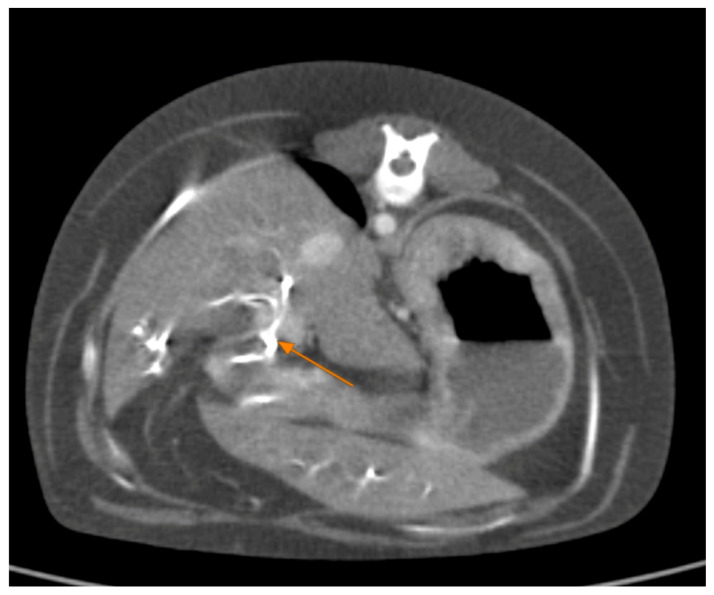
Abdominal contrast CT (axial plane). The common bile duct (outlined by orange arrows) is markedly dilated and packed with high-density barium sulfate.

Contrast-enhanced CT of the abdomen performed 48 h into the investigation, confirmed the presence of high-density material (barium sulfate) within the intrahepatic and extrahepatic biliary ducts. The CT also provided detailed information about the pancreas: the pancreatic parenchyma was irregularly enlarged and heterogenous, with regions of mottled hypoattenuation. Mild peripancreatic fat stranding (inflammation of surrounding fat) was evident, and multiple ill-defined, round hypoattenuating areas were present within the pancreas, consistent with abscesses or pockets of necrosis (Figure 5 and Figure 6). The common bile duct was markedly distended and filled with hyperattenuating contrast material (barium), confirming duodenobiliary reflux in cross-sectional detail (Figure 7).

**Figure 8:**
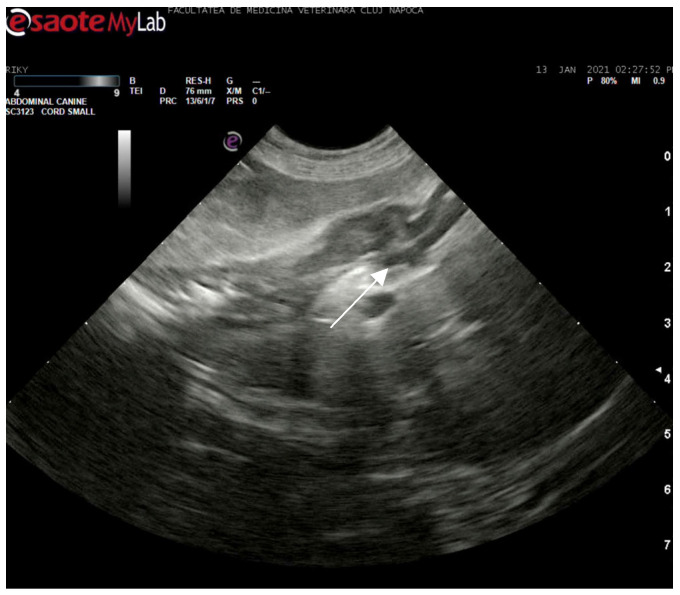
Ultrasonographic image of the duodenum and pancreas. There is severe duodenal wall thickening and a prominently distended major duodenal papilla (white arrow), consistent with inflammation and functional obstruction at the papilla. Surrounding the area is hyperechoic mesenteric fat due to pancreatitis.

**Figure 9:**
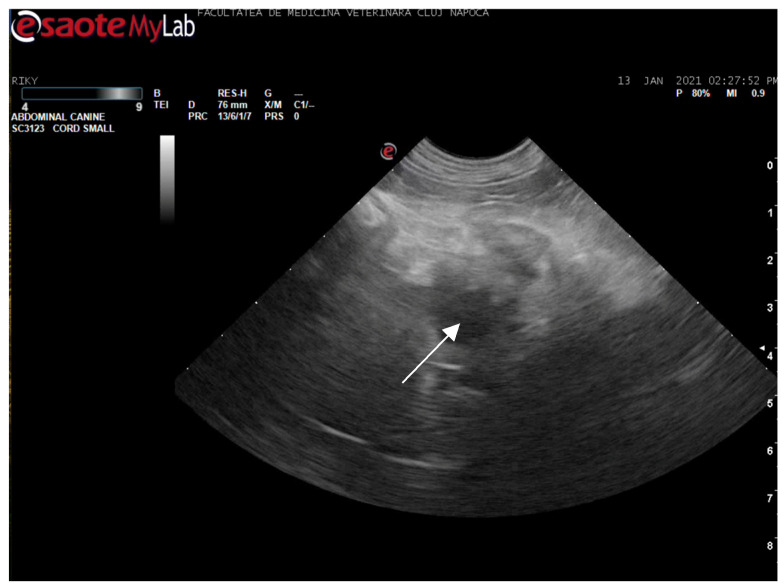
Ultrasonographic image of the pancreas revealing multiple oval hypoechoic lesions (abscesses) within the pancreatic parenchyma (white arrow) and hyperechoic peritoneal stranding. Fibrous adhesions between the pancreas and duodenum are present, which were confirmed at surgery.

Abdominal ultrasonography corroborated the radiograph and CT findings. The pancreas was enlarged, presenting hyperechoic parenchyma with several encapsulated hypoechoic lesions, measuring several millimeters, consistent with pancreatic abscesses or necrotic collections. The surrounding mesentery was hyperechoic, indicating focal peritonitis. In addition, hyperechoic mottled tissue between the pancreas and adjacent duodenum suggested adhesions. The duodenal wall was mildly thickened, and the major duodenal papilla was enlarged and appeared patulous (open). When imaged, the papilla region showed a small amount of echogenic fluid content. These sonographic findings indicated severe pancreatitis with abscessation and confirmed a widely open papilla, which could facilitate reflux. The exploratory laparotomy findings on day 3 (multiple pancreatic abscesses and fibrous adhesions) corresponded with the imaging observations (Figure 8 and Figure 9).

## 4. Discussion

This case represents the second documented instance of spontaneous duodenobiliary reflux in a dog, distinct from prior reports tied to intestinal obstruction [9] or enteroanastomosis [8]. In the present case, abscessing pancreatitis—a severe form of pancreatitis with abscess formation—appears to have been the precipitating cause of reflux. We hypothesize that inflammation and swelling of the pancreas, as well as the formation of pancreato-duodenal adhesions, led to dysfunction or compromise of the sphincter of Oddi. Inflammation of the sphincter area can impede its normal function or patency, effectively allowing duodenal contents (including gas, bile, and in this case barium) to flow backward into the biliary tree [12]. Transient sphincter of Oddi incompetence has been observed in human acute pancreatitis, contributing to duodenopancreatic or duodenobiliary reflux in about 12% of acute cases [13]. The current report is the first in veterinary medicine to link pancreatitis (specifically with abscesses) to spontaneous duodenobiliary reflux.

Historical and experimental evidence in the literature supports a close interplay between duodenal content reflux and pancreatitis. In 1909, Opie and Meakins documented a human autopsy case where reflux of duodenal contents into the pancreatic duct was postulated to have caused acute hemorrhagic pancreatitis [14]. Subsequent experimental studies have corroborated this mechanism: creation of a closed duodenal loop in dogs (thereby raising intraluminal duodenal pressure and forcing intestinal fluids into the pancreatic duct) reliably produced acute necrotizing pancreatitis [15]. These classic dog experiments demonstrated that obstructing the outflow of the duodenum can lead to reflux of duodenal enzymes and activate pancreatic autodigestion, a finding that has heavily influenced the understanding of pancreatitis pathogenesis. More recently, Isogai et al. (2024) revisited the duodenal reflux theory in the context of gallstone pancreatitis, delineating a “pancreatic-type” gallstone pancreatitis in which transient impaction of an ampullary stone allows duodenal (and biliary) reflux into the pancreatic duct, resulting in pancreatic necrosis [16]. While our case involves the reverse situation (pancreatitis causing reflux into the biliary system), these human studies underscore how a loss of the normal one-way control at the duodenal papilla can lead to pathological two-way traffic between the pancreatic/biliary ducts and the duodenum.

Unlike human cases of duodenobiliary reflux, where reflux is often associated with bacterial contamination of the biliary tract or gallstone formation when the reflux is chronic [4,17], our canine patient did not develop immediate complications such as ascending cholangitis or cholelithiasis from the reflux. The absence of such complications in this dog may be attributable to the short duration of reflux (transient event during acute illness) and rapid barium clearance from duodenum and biliary system, due to species-specific biliary dynamics [18]. Species-specific differences in biliary anatomy and physiology could also play a role; dogs may be less prone to persistent bile infections under these circumstances, or the event was simply too acute to allow colonization or stone formation. Human studies report duodenobiliary reflux rates as high as 35–66% in patients with T-tube biliary drainage or biliary stents (situations that disrupt normal papillary function) [12,17,19]. By contrast, spontaneous reflux appears exceedingly rare in dogs, suggesting that healthy canines have a very competent sphincter mechanism and that it requires severe pathology (such as pancreatitis in this case) to override it.

Barium sulfate proved invaluable for visualizing the biliary tree [6,7], with no adverse effects noted in our case, though human studies caution against prolonged retention risks [4,17]. In our patient, serial imaging confirmed that the barium was mostly eliminated over a few days. An incidental finding on the initial radiograph was pneumocholecystitis (gas in the gallbladder and biliary ducts). This could have arisen from gas-forming bacteria related to pancreatitis or from duodenal gas translocating due to papillary dysfunction. Its exact significance remains unclear, but it did alert us to a biliary abnormality prior to the administration of barium.

Potential differential diagnoses for the imaging findings (before the exploratory surgery confirmed pancreatitis) included biliary tract infection, papillary stenosis, or even an upper gastrointestinal perforation leading to a biliary fistula. Hepatobiliary neoplasia was considered less likely given the acute presentation and multifocal nature of the lesions. The multimodal imaging approach (combining radiographs, CT, and ultrasound) was critical in narrowing the differentials. It allowed us to confirm the presence of duodenobiliary reflux and to identify the pancreatic abscesses as the likely cause, aligning our diagnostic process with standards reported in human medicine for obscure biliary conditions [13,20].

Limitations: This report is limited by the lack of direct functional testing of the sphincter of Oddi (such as manometry or cholangiography) which might have definitively demonstrated sphincter incompetence [12]. Additionally, no histopathology or culture was obtained from the pancreatic abscesses, so the precise etiology of pancreatitis (e.g., infectious vs. sterile necrotizing pancreatitis) remains speculative. The patient’s euthanasia shortly after diagnosis meant we could not observe longer-term outcomes, such as whether chronic biliary damage, stricture, or recurrent infection would have occurred. Finally, as the second reported case in dog and third report of this pathology in animals [8,9], this case presentation can only suggest an association between pancreatitis and biliary reflux in dogs; further reports or studies would be needed to determine how frequently, if at all, pancreatitis leads to duodenobiliary reflux in the canine population.

## 5. Conclusions

This second reported canine case of spontaneous duodenobiliary reflux was linked to abscessing pancreatitis, a novel association in veterinary medicine. The presentation and outcome in this dog contrast with human duodenobiliary reflux cases, where complications like cholangitis are more commonly observed [1,2,13,17]. Multimodal imaging was pivotal for diagnosis in our case, confirming the reflux and uncovering its underlying cause. Our findings suggest that severe pancreatitis, especially with abscess formation, should be considered as a potential cause of duodenobiliary reflux in dogs. Recognition of this rare phenomenon is important, as it expands the differential diagnoses for biliary tree gas or contrast filling on imaging. Further investigation into species-specific mechanisms and long-term effects of duodenobiliary reflux in veterinary patients is warranted.

## Data Availability

The original contributions presented in this study are included in the article. Further inquiries can be directed to the corresponding author.

## Abbreviations

The following abbreviations are used in this manuscript:

GIT	Gastro-intestinal tract
ERCP	Endoscopic retrograde cholangiopancreatography
CT	Computed Tomography
BCS	Body Condition Score
CMPS-SF	The short form composite measure pain score
